# Within-Person Variation of Affective Well-Being during and after Exercise: Does the Person–Exercise Fit Matter?

**DOI:** 10.3390/ijerph18020549

**Published:** 2021-01-11

**Authors:** Julia Schmid, Vanessa Gut, Nina Schorno, Takuya Yanagida, Achim Conzelmann

**Affiliations:** 1 Institute of Sport Science, University of Bern, 3012 Bern, Switzerland; vanessa.gut@ispw.unibe.ch (V.G.); nina.schorno@ispw.unibe.ch (N.S.); achim.conzelmann@ispw.unibe.ch (A.C.); 2 Department of Developmental and Educational Psychology, Faculty of Psychology, University of Vienna, 1010 Vienna, Austria; takuya.yanagida@univie.ac.at

**Keywords:** enjoyment, affect, motivation, preferences, perceived competence, physical activity

## Abstract

Affective well-being is positively linked to regular exercise. Therefore, it is important to identify the factors that influence intra-individual variability of affective well-being. This study investigated (1) whether affective responses vary within an individual and (2) how affective responses are associated with a motive–incentive fit and a skill–task fit. A total of 107 adults (66% females, *M*_age_ = 41.79 years old, 58% doing no exercise) took part in three exercise sessions in a random order. Each session lasted 30 min with a break of 10 min between. The sessions were similarly structured but covered diverse activity incentives (e.g., figure vs. social contact vs. aesthetic movements). Intraclass correlation coefficients showed a very high within-person variation of affective valence and enjoyment across the exercise sessions. The results of multi-level regression analyses revealed that associations between perceived competence, considered to be an indicator of the skill–task fit, and affective well-being were moderate to high, whereas those between motive–incentive fit and affective well-being were low to moderate.

## 1. Introduction

Affective well-being plays an important role in initiating and maintaining regular exercise. According to hedonistic theory, many humans seek pleasure [1]. In fact, studies show that affective well-being during exercise is positively associated with motivation and future behaviour [2,3].

In the last decades, research has increasingly drawn attention to the variability of affective well-being during and after exercise. Most studies have focused on inter-individual variability and have used a single exercise session design [4], whereas intra-individual variability over multiple sessions has rarely been examined. However, Unick et al. [5] demonstrated that intra-individual differences in affective responses to exercise are, indeed, substantial, even for standardised exercise sessions. They investigated affective responses to three treadmill bouts that were performed at the same intensity, for the same duration, and at the same time of day. 24–30% of the variability in affective well-being were due to within-person differences. Jeckel et al. [6], in contrast, examined self-chosen activities in everyday life. In their study, affective well-being varied much more intra-individually, at 59% (see also [7,8]).

To develop interventions that promote exercise behaviour, it is essential to identify the factors that influence intra-individual variability of affective well-being [9]. One such influencing factor may be the person–exercise fit [10]. Fit theory claims that a good match between people’s characteristics and their environment results in positive outcomes, such as well-being [11]. The fit theory is firmly rooted in the tradition of Kurt Lewin’s maxim that behaviour is a function of person and environment [12]. It is tested mainly in organisational psychology (e.g., person–vacation fit [13]), however, fit theory has also been examined in the field of sport and exercise. For example Schüler et al. [14] and Sudeck et al. [15] demonstrated that a fit between a person’s motives and goals (e.g., social contact) and the incentives provided by an exercise activity (e.g., having the opportunity to socialize with others while doing partner exercises) results in positive affective well-being. Furthermore, a fit between a person’s skills (e.g., crawl technique mastery) and the demands of the task (e.g., to swim 500 m) is also beneficial; if a person feels competent during exercise, this promotes affective well-being [16,17,18].

This study investigates (1) whether affective well-being during and after multiple exercise sessions vary within an individual and (2) how affective well-being during and after exercising is influenced by both motive–incentive and skill–task fits. Compared to current research, this focus is noteworthy due to the two further steps taken: Firstly, the present study took place in a non-laboratory setting. This allowed features to be measured in closer adherence to naturalistic conditions than has been done in previous studies with controlled laboratory conditions [14]. Secondly, affective well-being is measured in both a categorical and dimensional way. According to the categorical approach of affect, affective states are organized into distinct categories, such as enjoyment [3]. According to the dimensional approach, affective states are systematically interrelated, therefore they can only be described using a few basic affect dimensions. One such dimension is affective valence, defined as the degree of pleasure or displeasure [19]. Measuring affective well-being in these two complementary ways allows a deeper understanding of the relationship between affective well-being and person–exercise fit within individuals.

## 2. Materials and Methods

### 2.1. Participants

The data of this study were collected in summer 2019 within the scope of two exercise counselling events. The events were organised by the University of Bern in cooperation with a health insurance company. They targeted inactive and moderately active people (doing sport or exercise for 0–120 min/week), who were between the ages of 20 and 70. The events were advertised via a health insurance’s online media (newsletter, posts in social media) and print media (magazine, flyer, media release). Interested persons were randomly assigned to one of the two event dates. In total, 107 people (66% females, *M*_age_ = 41.79 years old, range: 20–66 years, 58% doing no exercise) were included in this study. Table S1 in the Supplementary Materials shows the detailed sample characteristics. All participants provided their informed written consent to the investigation.

### 2.2. Procedure and Intervention

After participants were welcomed and informed about the course of the study, they filled out an online survey to assess their background characteristics, motives and goals for exercise. Following this, three trial exercise sessions were held consecutively. They lasted 30 min with a break of 10 min between each session. They were similarly structured (warm-up, main part, cool down) but covered diverse activity incentives (see Table 1 and Figure S1 in the Supplementary Materials). Participants did not know what to expect in the sessions, as they were labelled neutrally A, B and C. They were randomized in one of six sequences (e.g., ABC, ACB, BAC, BCA and so on). Sequences were completely counterbalanced. The trial exercise sessions were held by three trained instructors in small groups of 8–12 people. The sessions were scripted and therefore standardised. To keep the teaching climate as constant as possible across all trial sessions, instructors did not encourage or motivate individually. The training intensity should be moderately high across all main parts to ensure this, participants could choose an individual intensity suitable to them between two levels of difficulty in the cardio and strength exercises (session A). Furthermore, group games were not played with running speed, but in fast walking to avoid intensity peaks (session B), and dance activities included whole body movements (session C). Instructors told participants in every session to do the exercises in an intensity that they can carry on a conversation comfortably. In each session, participants used smartphones to evaluate their incentives, perceived competence, affective valence and enjoyment. The ethics commission of the Faculty of Human Sciences of the University of Bern approved the study design and procedures (number: 2018-11-00004).

### 2.3. Measures

#### 2.3.1. Perceived Competence

The skill–task fit was measured via perceived competence directly after each exercise trial session. The single item was phrased with reference to the flow state scale [20]. People had to rate the statement “I felt very competent during the physical activity session” on a 7-point scale from 1 (not at all true) to 7 (completely true).

#### 2.3.2. Motives and Goals for Exercise

Motives and goals were assessed before the start of the exercise trial sessions, using the Bernese Motive and Goal Inventory (BMZI). The BMZI has a good validity and reliability both for less active and more active adults [21]. Participants were asked the following question: «Why do you exercise/why would you exercise?» The BMZI consists of 23 items and covers the following seven dimensions: Contact (e.g., «to get to know people »), Competition/Achievement (e.g., «to compete with others»), Distraction/Catharsis (i.e., «to reduce stress»), Figure/Appearance (e.g., «to regulate my weight»), Fitness (e.g., «to keep myself in good physical shape»), Health (e.g., «to improve my state of health») and Aesthetics (e.g., «for enjoyment of beautiful movements»). Each item is accompanied by a 5-point response scale from 1 (I strongly disagree) to 5 (I strongly agree). The internal consistency of the subscales was good (0.745 ≤ α ≤ 0.831).

#### 2.3.3. Perceived Incentives in Exercise

Incentives were recorded directly after each exercise trial session in accordance with the BMZI. Individuals had to rate what they experienced during the exercise activities. Every dimension of the BMZI was assessed with one or two marking items: Contact (e.g., «I got to know people»), Competition/Achievement (e.g., «I competed with others»), Distraction/Catharsis (i.e., «I was able to reduce stress»), Figure/Appearance (e.g., «I did something to regulate my weight»), Fitness (e.g., «I did something to keep myself in good physical shape»), Health (e.g., «I did something to improve my state of health») and Aesthetics (e.g., «I experienced beautiful movements»). The response format was again a 5-point scale from 1 (I strongly disagree) to 5 (I strongly agree). The mean value was used for statistical analysis when more than one item represented the area. The internal consistencies of the subscales were between 0.554 ≤ α ≤ 0.907.

#### 2.3.4. Affective Valence

Affective valence was assessed during (between minutes 12–14) and directly after each trial session, using a German version of the Feeling Scale (FS) [22,23]. High correlation coefficients with a parallel test indicate a good validity of the translated FS [23]. Individuals have to rate how they currently feel on a single item scale, with answer options ranging from +5 (very good) to −5 (very bad) and with a neutral answer possibility.

#### 2.3.5. Exercise Enjoyment

Enjoyment was assessed using the validated single–item Exercise Enjoyment Scale (EES) [24] directly after each exercise session. The EES utilises the stem, «Use the following scale to indicate how much you enjoyed this exercise session». People had to pinpoint their answer on a continuous 100 mm scale, with not at all at one end and extremely at the other.

### 2.4. Data Preparation and Analyses

To examine the motive–incentive fit, new variables were calculated by subtracting motive values from incentive values for each person in each exercise trial session (e.g., session A: Contact_motive_ − Contact_incentive_; session B: Contact_motive_ − Contact_incentive_, and so on). Negative values were recoded as positive. Low values in these variables mean that the difference between motive and experienced incentive is small and therefore the fit in the session under consideration is good. To simplify the analysis, only the three most important motives for each individual (e.g., Contact, Fitness, Figure/Appearance) were considered and their mean motive–incentive fit was included (e.g., session A: Mean [Contact_motive_ − Contact_incentive,_ Fitness_motive_ − Fitness_incentive,_ Figure/Appearance_motive_ − Figure/Appearance_incentive_]).

To examine whether affective well-being varied within an individual across the three exercise trial sessions, intraclass correlation coefficients (ICC) ($\mathrm{ICC}=\frac{\sigma^{2}\;\mathrm{between}\;\mathrm{persons}}{\left(\sigma^{2}\;\mathrm{within}\;\mathrm{person}\;+\;\sigma^{2}\;\mathrm{between}\;\mathrm{groups}\;+\;\sigma^{2}\;\mathrm{between}\;\mathrm{persons}\right)}$) were calculated and subtracted from 1. Multi-level regression models were used to analyse whether perceived competence and a motive–incentive fit are associated with affective well-being during and after exercise. These models take into account the nested data structure (Level 1: exercise sessions, Level 2: individuals) resulting from the within-subject research design. Separate models were calculated for valence during, valence after and enjoyment after exercise. All models were calculated as random intercept-only models and estimated using robust maximum likelihood method. The group membership was accounted for by using cluster-robust standard errors. Participant’s age and gender were integrated as control variables. The perceived competence and the motive–incentive fit were centered around the group mean [25]. The alpha level of the tests was set to *p* < 0.05. All analyses were done with Mplus 8 (Muthén & Muthén, Los Angeles, CA, USA) [26].

## 3. Results

Descriptive statistics of the study variables can be found in the Supplementary Materials in Table S1 and within-person correlation coefficients in the Supplementary Materials in Table S2. While the variability of affective valence and enjoyment between persons was relatively low, reliability within persons was relatively high. The ICCs revealed that 89% (valence during exercising), 67% (valence after exercising) and 88% (enjoyment after exercising) of the total variability were due to within-person differences (see Table 2).

The results of the multi-level regression analyses are presented in Table 3. The control variables age and gender were not linked with indicators of affective well-being. However, perceived competence was positively related to affective valence and enjoyment (0.189 ≤ β ≤ 0.469). The motive-incentive fit was also associated with valence and enjoyment (−0.206 ≤ β ≤ −0.157). The negative β-coefficients of the variable “Motive-incentive fit” mean that the smaller the difference between a person’s three most important motives and the corresponding incentives, the higher their affective well-being. The independent variables could explain 16% of the within-variance of affective valence during exercising and 8% of the within-variance of affective valence after exercising. In contrast, the explained variance in enjoyment was a lot higher, at 31%.

## 4. Discussion

The aim of this study was to examine whether affective responses during and after three exercise sessions vary within an individual and how a person-exercise fit is associated with affective responses. The study results showed very high intra-individual variances, both in valence and enjoyment. Compared to existing studies which investigated affective response across self-chosen activities [6] or across similar/identical exercise sessions [5,7], the calculated ICCs were notably low. In the present study, intra-individual variability of valence was higher during exercise than after. This pattern may be partly caused by a robust, positive rebound-effect post-exercise across sessions [17]. People felt, for instance, similarly relieved after each exercise session.

In the present study, person-exercise fit was related to affective well-being during and after exercise. However, associations between perceived competence, considered to be an indicator of the skill-task fit, and affective well-being were moderate to high, whereas those between motive-incentive fit and affective well-being were low to moderate. This could be due to the fact that participants in the study often had extrinsic motives, such as Figure/Appearance, which lead to fewer changes in well-being than more intrinsic motives, such as Aesthetics [27]. Furthermore, it was noticed that variables for person-exercise fit were more closely related to enjoyment than to affective valence. With its explicit reference to the exercise session, enjoyment might have a stronger evaluative component than valence.

This study was strengthened by its repeated assessments across diverse exercise sessions in natural surroundings. Thus, the present research has high ecological validity. Nevertheless, it has some limitations. Firstly, the measurement of person-exercise fit must be critically viewed and discussed. The calculated fit index is based on absolute values and therefore conceals the direction of motive-incentive discrepancies [11]. In contrast to the motive-incentive fit, the skill-task fit was measured via people’s perceived competence. These different operationalisations may have influenced the results [11]. Secondly, the intensity of exercise was not objectively standardised across sessions, although it plays an important role in affective responses. According to the Dual-Mode theory, aspects of the person–exercise fit may have a particularly strong influence during vigorous intensities [17]. Thirdly, carry-over effects may have influenced our study results (e.g., affective well-being in a previous exercise session influenced affective well-being in a subsequent exercise session). Although the use of random and counterbalanced sequence of the exercise sessions and the 10 min break between sessions helps to mitigate or control this risk, future studies need to use an optimized design. For instance, the wash out period should be extended by having a break of one or more days between exercise sessions. Fourthly, enjoyment was not assessed during exercise. However, to compare the effects of person-exercise fit on valence and enjoyment, the two variables should have always be assessed together. Finally, the age range of the sample was relatively wide. On the one hand, this can be seen as positive, because many studies are limited to university-aged participants [14]. On the other hand, with an age-mixed sample, it remains unclear whether the results found for the overall sample also apply to specific sub-samples. Future studies should not only control for age but consider it more systematically and replicate our results in a sample of younger or older adults.

## 5. Conclusions

In conclusion, the study demonstrated a high variability in affective well-being across three exercise sessions within inactive and moderately active people. Intra-individual differences were associated with a fit between a person’s motor skills and task demands, and with a fit between a person’s three most important motives and the activity incentives. Overall, the findings suggest that the person-exercise fit warrants more attention in research. Furthermore, future research should examine how it can be promoted. A possible intervention for fostering the motive-incentive fit could be an exercise and sport counselling, in which people are guided to reflect on their own motives and exercise experiences and thereby assisted in finding an exercise activity that suits them [28].

## Figures and Tables

**Table 1:** Structure and content of the three exercise trial sessions.

	Session A	Session B	Session C
Focused incentives	Figure/Appearance, Health	Contact, Competition/Performance	Aesthetics, Distraction/Catharsis
Warm-up 5 min	Dynamic stretching and gentle strengthening exercises, e.g., jumping jacks, high knees, squats with upper body rotation	Name game and ice-breaker with light running, e.g., participants had to find someone, who has the same hobby	Full-body mobilization with flowing movements, e.g., leg swings, easy body waves, hip circles
Main part 20 min	Cardio exercises, e.g., running/walking around doing lunge jumps when the instructor claps; core-strength exercises	Competitive group games with the ranking announced afterwards, e.g., ball tag, relay races with a hula hoop	Several dance activities with music, e.g., jazz and contemporary dance elements; Tai Chi exercises focusing on breathing and soft movements, e.g., rollback and push movements
Cool-down 5 min	Full-body stretching, e.g., hamstrings and lower back, neck and chest	Cooperative game, e.g., the whole group stands in a circle holding hands, one half leans forward the other half backward	Body awareness exercises, in standing, with eyes closed, e.g., feeling different parts of the body on a mental journey

**Table 2:** Descriptives of the time-varying variables: Intraclass correlation coefficients, means, and variance components.

Variables (Range)	ICC	Between Persons-Level		Within Person-Level
		*M*	*ơ* ^2^	*ơ* ^2^
Perceived competence (1 to 7)	0.295	4.903	0.601	1.417
Motive–incentive fit (0 to 4)	0.211	0.963	0.084	0.261
Affective valence during session (−5 to +5)	0.107	3.062	0.318	2.619
Affective valence after session (−5 to +5)	0.322	3.654	0.597	1.249
Exercise enjoyment after session (0 to 100)	0.188	69.558	64.050	234.294

Notes: Motive-incentive fit = calculated motive-incentive fit for the three most important motives; *ơ*^2^ = variance.

**Table 3:** Within person-associations between perceived competence, motive–incentive fit and affective well-being during and after exercise sessions (*n* = 107).

	Affective Valence during Exercise				Affective Valence after Exercise				Enjoyment after Exercise			
	*B*	*SE*	*β*	*p*	*B*	*SE*	*β*	*p*	*B*	*SE*	*β*	*p*
Age (control variable)	0.009	0.006	0.153	0.112	0.002	0.010	0.031	0.835	0.151	0.117	0.170	0.198
Gender (control variable)	0.283	0.158	0.191	0.128	0.007	0.180	0.004	0.967	1.357	2.457	0.059	0.581
Perceived competence	0.501	0.050	0.312	<0.0005	0.214	0.054	0.189	<0.0005	8.000	1.064	0.469	<0.0005
Motive–incentive fit	−0.662	0.069	−0.177	0.011	−0.413	0.083	−0.157	<0.0005	−8.206	2.142	−0.206	<0.0005

Notes: Motive-incentive fit = calculated motive-incentive fit for the three most important motives; *β* = standardised regression coefficient.

## Data Availability

The data presented in this study are available on request from the corresponding author.

## Supplementary Materials

The following are available online at https://www.mdpi.com/1660-4601/18/2/549/s1, Table S1: Sample characteristics (*n* = 107) and descriptive statistics, Table S2: Within-person correlation matrix for all study variables (*n* = 107)*,* Figure S1: Perceived incentives in exercise session A, B and C (between person means; *n* = 107).
