# Supplementary material for: Within-Person Variation of Affective Well-Being during and after Exercise: Does the Person–Exercise Fit Matter?
Source: Int J Environ Res Public Health. 2021 Jan 11;18(2):549. doi: 10.3390/ijerph18020549 (PMC7826600; doi:10.3390/ijerph18020549)

Supplementary material

Table S1. *Sample characteristics (n = 107) and descriptive statistics*

**Variables**	**% or Mean (SD)**
Gender	66% females
Age	41.79 years (12.36 years); range: 20-66 years
Highest education	
Vocational education and training	25%
Bacccalaureate school	12%
University or university of applied science	58%
Others	5%
BMI	26.50 kg/m^2 (5.31 kg/m^2)
Normal weight (18 ≥ BMI ≤ 25)	41%
Overweight (25 > BMI ≤ 30)	41%
Obese (BMI > 30)	18%
Exercise behaviour (last 4 weeks)	51.73 minutes/week (88.08 minutes/week)
No exercise	58%
1-74 minutes	18%
≥ 75 minutes	24%
Motives and goals for exercise	
Distraction/Catharsis	2.98 (1.08)
Fitness	4.33 (0.59)
Health	4.13 (0.73)
Competition/Achievement	1.83 (0.82)
Aesthetics	2.54 (1.07)
Contact	2.41 (0.85)
Figure/Appearance	3.66 (1.06)

Table S2. *Within-person correlation matrix for all study variables (n = 107)*

	Affective valence after session	Affective valence during session	Perceived competence	Motive-incentive fit
Exercise enjoyment after session	0.426	0.232	0.440	-0.144
Affective valence after session		0.789	0.371	-0.143
Affective valence during session			0.779	-0.229
Perceived competence				-0.147

Figure S1. *Perceived incentives in exercise session A, B and C (between person means; n = 107)*

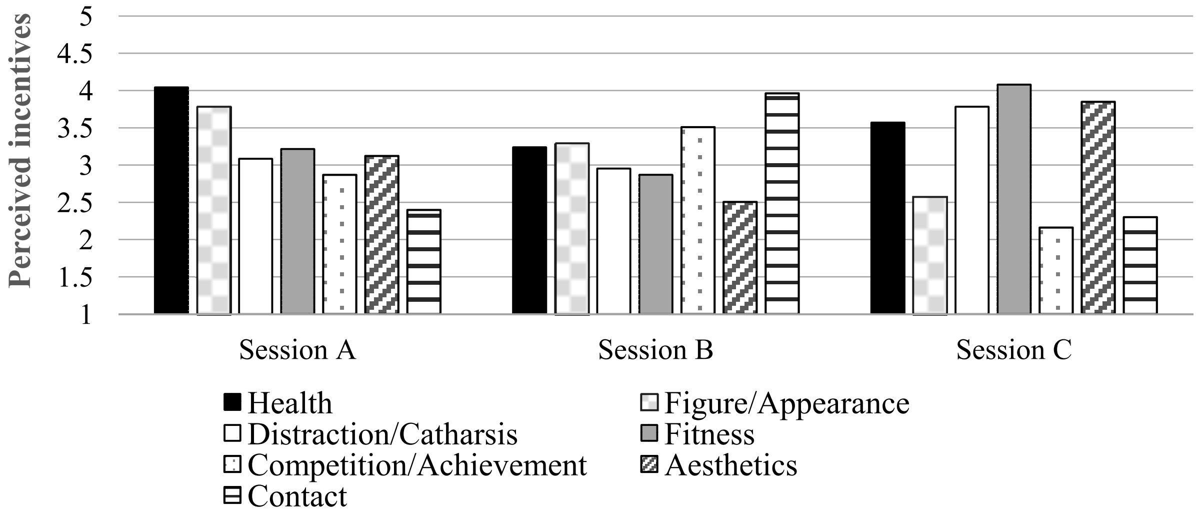

Supplement: Supplementary file 1 [file ijerph-18-00549-s001.pdf]
